# Supplementary material for: Subcutaneous ketamine infusion in palliative patients for major depressive disorder (SKIPMDD)—Phase II single-arm open-label feasibility study
Source: PLoS One. 2023 Nov 14;18(11):e0290876. doi: 10.1371/journal.pone.0290876 (PMC10645343; doi:10.1371/journal.pone.0290876)
Supplement: S1 Fig — Abbreviations: BPRS—Brief Psychiatric Rating Scale; CADSS—Clinician Administered Dissociative States Scale; MADRS—Montgomery-Asberg Depression Rating Scale; PHQ-2—Patient Health Questionnaire-2. *Baseline MADRS score is the MADRS score prior to the last ketamine dose (default) if relapse (MADRS of ≤9) has not occurred. If relapse has occurred, the MADRS score at relapse becomes the baseline. (DOCX) [file pone.0290876.s003.docx]

**S1 Figure. SKIPMDD Study Procedure [37]**


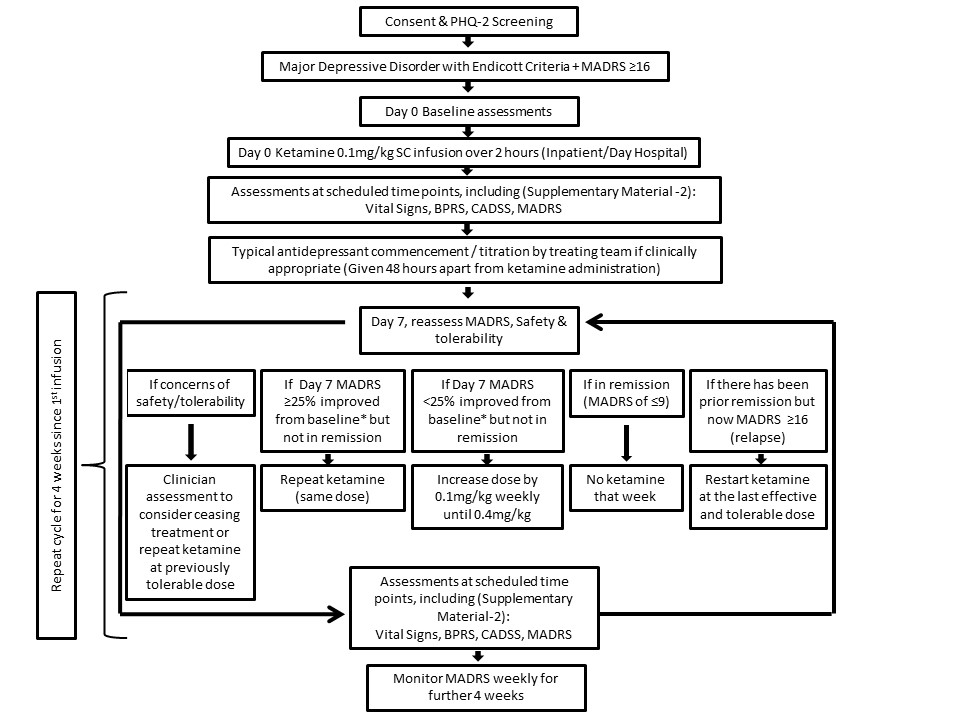


Abbreviations: BPRS - Brief Psychiatric Rating Scale; CADSS - Clinician Administered Dissociative States Scale; MADRS - Montgomery-Asberg Depression Rating Scale; PHQ-2 - Patient Health Questionnaire-2

*Baseline MADRS score is the MADRS score prior to the last ketamine dose (default) if relapse (MADRS of ≤9) has not occurred. If relapse has occurred, the MADRS score at relapse becomes the baseline.
