# Supplementary material for: Subcutaneous ketamine infusion in palliative patients for major depressive disorder (SKIPMDD)—Phase II single-arm open-label feasibility study
Source: PLoS One. 2023 Nov 14;18(11):e0290876. doi: 10.1371/journal.pone.0290876 (PMC10645343; doi:10.1371/journal.pone.0290876)
Supplement: S1 Table — Abbreviations: AKPS—Australia-modified Karnofsky Performance Scale; BPRS—Brief Psychiatric Rating Scale; CADSS—Clinician Administered Dissociative States Scale; ECG–Electrocardiogram; EUC–Electrolyte Urea Creatinine; FBC–Full Blood Counts; LFT–Liver Function Test; MADRS—Montgomery-Asberg Depression Rating Scale; NCI CTCAE—National Cancer Institute Common Terminology Criteria for Adverse Events; PHQ-2—Patient Health Questionnaire-2; TFT–Thyroid Function Test. (DOCX) [file pone.0290876.s004.docx]

**S1 Table.** **Assessment Schedule [37]**

| **Assessments** | **Eligibility** | **Baseline**  **(t0 min)** | **30 min** | **1hr** | **1.5hr** | **2hr**  **(infusion complete)** | **4hr** | **6hr** | **1 day** | **2 days** | **3 days** | **7 days** | **Weekly (day 7) if no repeat ketamine infusion**  **(up to 8 weeks from initial dose)** |
| --- | --- | --- | --- | --- | --- | --- | --- | --- | --- | --- | --- | --- | --- |
| **Informed consent** | **X** | **X**  (Re-affirm) |  |  |  |  |  |  |  |  |  |  |  |
| **PHQ-2** | **X** |  |  |  |  |  |  |  |  |  |  |  |  |
| **Endicott Criteria** | **X** |  |  |  |  |  |  |  |  |  |  |  |  |
| **AKPS** | **X** | **X** |  |  |  |  |  |  | **X** | **X** | **X** | **X** | **X** |
| **Vital Signs** |  | **X** | **X** | **X** | **X** | **X** | **X** | **X** |  |  |  |  |  |
| **ECG** |  | **X** |  |  |  |  |  |  |  |  |  |  |  |
| **Bloods (FBC/LFT/EUC /TFT)** | **X** |  |  |  |  |  |  |  |  |  |  |  |  |
| **MADRS**[33] | **X** | **X** |  |  |  |  |  | **X** | **X** | **X** | **X** | **X** | **X** |
| **BPRS**[43, 44] |  | **X** |  |  |  | **X** | **X** | **X** |  |  |  |  |  |
| **CADSS**[45, 46] |  | **X** |  |  |  | **X** | **X** | **X** |  |  |  |  |  |
| **Adverse Events**  **(NCI CTCAE 4.03)**[47] |  | **X** | **X** | **X** | **X** | **X** | **X** | **X** | **X** | **X** | **X** | **X** | **X** |
| **Concomitant medications** |  | **X** |  |  |  |  |  |  |  |  | **X** | **X** | **X** |

Abbreviations: AKPS - Australia-modified Karnofsky Performance Scale; BPRS - Brief Psychiatric Rating Scale; CADSS - Clinician Administered Dissociative States Scale; ECG – Electrocardiogram; EUC – Electrolyte Urea Creatinine; FBC – Full Blood Counts; LFT – Liver Function Test; MADRS - Montgomery-Asberg Depression Rating Scale; NCI CTCAE - National Cancer Institute Common Terminology Criteria for Adverse Events; PHQ-2 - Patient Health Questionnaire-2; TFT – Thyroid Function Test
