# Supplementary material for: Subcutaneous ketamine infusion in palliative patients for major depressive disorder (SKIPMDD)—Phase II single-arm open-label feasibility study
Source: PLoS One. 2023 Nov 14;18(11):e0290876. doi: 10.1371/journal.pone.0290876 (PMC10645343; doi:10.1371/journal.pone.0290876)
Supplement: S2 Table — (PDF) [file pone.0290876.s005.pdf]

CONFIDENTIAL

CASE REPORT FORM

033 SKIPMDD

Consent Capacity Form

This Case report form is to be completed by the principal investigator or medical delegate in compliance with PaCCSC Standard Operating Procedures.

This form is used by at the consenting stage, alongside with the Participant Informed Consent Form, to assess the potential participant's capacity to consent in the context of medical illness and depression.

Signature of Principal Investigator or Medical Delegate _________________

**Initials**	**Date**

Forms required for this visit:

PICF

Procedure:

To start, the principal investigator or medial delegate will explain to the potential participant the need for assessing his/her capacity to consent, using the below statement as a guide:

"Mr/Mrs/Ms XXX, thank you for considering this trial. Sometimes when patients get sick, it can affect the thinking and ability to understand information. This can lead us to make decisions that we don't mean to make. To make sure that you are making a well-informed decision, we will be looking into your ability to make decisions about participating in this trial whilst we explain this trial to you."

The Participant Information Consent Form, alongside with all the relevant information about the SKIPMDD trial, will now be explained to the potential participant.

To proceed with signing the PICF, the potential participant must demonstrate competency (having the capacity) to consent to this SKIPMDD trial. This is demonstrated by showing that the potential participant is able to meet all of the following criteria during the interview with research team (tick all when checked to show competency):

Domains of Informed Consent	***Yes***	***No***
**1. Understanding**		
Understanding of disclosed information about the nature of the research project and its procedures in relation to the aim, the treatment, the potential benefits and risk about participating in the study, and understand that he/she can refuse to participant anytime and still receive ordinary care		

**2. Appreciation**		
Determine whether potential participants can acknowledge /appreciation how he or she will be affected by the decision to participate in the research project.		
i. Person maintains he or she is being recruited for a reason related only to <u>potential</u> personal benefit		
ii. Person provides response consistent with the idea that the research protocol, not personal preferences, will determine the experimental condition to which he or she will be assigned (i.e. If the participant is in remission, he/she will not get a ketamine dose that week until further review even if he/she would like to)		
**3. Reasoning**		
Reasoning in the process of deciding about participation, focusing on individual's ability to compare alternatives in light of their consequences		
i. Person can mention the consequences for participating and not participating in the trial, comparing between them the choices, and know how making the choice might influence their everyday life (e.g. endure the inconvenience of being followed up at a set schedule)		
**4. Expressing or evidencing a choice**		
Expression a choice about research participation which follows logically from the subject's own reasoning with consistency		

Final outcome:

Is the potential participant assessed to be competent / having the capacity to consent?

☐ Yes → proceed with signing PICF if potential participant agrees, and continue with the screening process using Patient Health Questionnaire -2 (PHQ-2)

☐ No → principal investigator or medical delegate inform participant that he or she cannot proceed with the study, stating:

> "Mr/Ms/Mrs XXX, despite how much we would love you to be on this trial, I am sorry to let you know that we won't be able to do so. This is because, I don't think you are in a good enough state right now to make decision about participating in this trial. We can always re-assess when you are better. Meanwhile, this doesn't mean there won't be anyone to look after your emotional wellbeing. We will let your treating doctors know to make sure that you continue to get the best care possible."

Investigator Signature	
Investigator name	
Date	
