# Supplementary material for: Subcutaneous ketamine infusion in palliative patients for major depressive disorder (SKIPMDD)—Phase II single-arm open-label feasibility study
Source: PLoS One. 2023 Nov 14;18(11):e0290876. doi: 10.1371/journal.pone.0290876 (PMC10645343; doi:10.1371/journal.pone.0290876)
Supplement: Attachment — Submitted filename: renamed_ca8a8.docx [file pone.0290876.s006.docx]

# COMMENTS – Manuscript PONE-D-23-04868

Title: “Subcutaneous Ketamine Infusion in Palliative Patients for Major Depressive Disorder (SKIPMDD)”

**Important note: This review pertains only to ‘statistical aspects’ of the study and so ‘clinical aspects’ [like medical importance, relevance of the study, ‘clinical significance and implication(s)’ of the whole study, etc.] are to be evaluated [should be assessed] separately/independently. Further please note that any ‘statistical review’ is generally done under the assumption that (such) study specific methodological [as well as execution] issues are perfectly taken care of by the investigator(s). This review is not an exception to that and so does not cover clinical aspects {however, seldom comments are made only if those issues are intimately / scientifically related & intermingle with ‘statistical aspects’ of the study}. Agreed that ‘statistical methods’ are used as just tools here, however, they are vital part of methodology [and so should be given due importance]. I look at the manuscript in/with statistical view point, other reviewer(s) look(s) at it with different angle so that in totality the review is very comprehensive. However, there should be efforts from authors side to improve (may be by taking clues from reviewer’s comments). Therefore, please do not limit the revision only (with respect) to comments made here.**

**COMMENTS:** Although this manuscript is well drafted [and the study is excellent with respect to most of the aspects], I have few observations/concerns (different opinion) which are given below:

Since according to ‘Methods’ section in ‘Abstract’, “This was a single arm, open-label, phase II feasibility study” a mention of this may appear in title {presently it gives the impression that it’s a full-fledged phase-III trial}. Very good it is clearly stated in the ‘Abstract’ itself, such a mention in title is desirable, I guess.

I am sure, authors are well aware of ‘single arm/group’ study, however, I request them to read a note pasted from one famous standard textbook on ‘Medical Research Methodology’:

It is very essential to keep the limitations in mind while interpreting results from any ‘single arm/group’ study. A classical/ideal clinical trial/study needs/requires a concurrently {but similarly} handled/treated appropriately selected/chosen control/comparison parallel group/arm. Note further that “Inferential statistics (i.e., hypothesis testing + estimation of CI) is built on the population model [which means the underlying assumption is that there is/are population(s) and we are dealing with random sample(s) drawn from that/those population(s)]. Although in clinical trial (involving at least two groups) we do not really deal with random samples (generally a non-probabilistic convenience sampling), ‘allocation’ to treatment groups is ‘randomly’ done which enable us to evoke the population model and we can use inferential statistics safely. But when there is only one group (so that there is no question of random allocation), with ‘non-random’ selection, it may be questionable to use inferential statistics even if you have two measurement sets as ‘pre-post’ or many repeated measurements or use ‘internal grouping for comparison.”

Except these minor points, the article is acceptable. Since ‘when the study is ‘pilot’ in nature, many things are ignored [loosely looked at / evaluated] {example: sample size is not a big issue, other methodology issues need not be looked at very rigorously, etc.’ However, mind you that as pointed out in ‘important note’ above “This review pertains only to ‘statistical aspects’ of the study and so ‘clinical aspects’ should be assessed separately/independently. ‘Minor Revision’ is recommended.
