# Supplementary material for: Subcutaneous ketamine infusion in palliative patients for major depressive disorder (SKIPMDD)—Phase II single-arm open-label feasibility study
Source: PLoS One. 2023 Nov 14;18(11):e0290876. doi: 10.1371/journal.pone.0290876 (PMC10645343; doi:10.1371/journal.pone.0290876)
Supplement: Attachment — Submitted filename: Response to Reviewers PLoS One 2 v1.docx [file pone.0290876.s007.docx]

Editor-in-Chief

PLoS One

Re: “Subcutaneous Ketamine Infusion in Palliative Patients for Major Depressive Disorder (SKIPMDD)” (PONE-D-23-04868R1)

Thank you for the opportunity to revise this paper.

Each of the issues raised has been addressed in the order in which it appeared in the editor’s letter. Attached are clean and track changed versions of the manuscript.

|  | **Editor’s/Reviewers’ comments** | **Authors’ responses** |
| --- | --- | --- |
| **Editor’s Comments**  We've checked your submission and before we can proceed, we need you to address the following issues: | | |
|  | 1. Please amend the title either on the online submission form or in your title page so that they are identical. | Title has now been amended in the title page of the manuscript v1.9, in line with the online submission platform. |
|  | 2. We note your current Competing Interests Statement: "CS is the recipient of the Sydney Partnership for Health, Education, Research and Enterprise (SPHERE) Palliative Care Clinical Academic Group Seed Grants. RC receives consulting fees or payments from Cymra Life Sciences Limited, Tilray Australia and New Zealand, AstraZeneca Pty Limited, A. Menarini Australia Pty Ltd. RC also receives payments for expert testimony from Office of the Director of Public Prosecutions NSW. AB assists in running a tertiary referral ketamine service at Black Dog Institute, running the Spravato (intranasal esketamine) early access program, and acting as a site principal investigator on a related quality of life study. CL participated in Janssen Advisory Board, acted as an unpaid consultant for Douglass Pharmaceuticals, and received support from Royal Australian and New Zealand College of Psychiatrists (RANZCP) for presenting at RANZCP Congress. DC is a paid consultant and advisory board member for Helsinn Pharmaceuticals, a paid consultant for Mayne Pharma International Pty Ltd, a paid subcontractor for Nous Group Pty Ltd, a paid board member for Icare Dust Diseases Board, and unpaid consultants for Chris O’Brien Lifehouse and Illawarra Health and Medical Research Institute (IHMRI). DC also receives payment from Mayne Pharma International Pty Ltd for intellectual property. Other authors declare no conflict of interest."  In order to provide our readers with as much transparency as possible, PLOS ONE policy requires that certain statements be present in the Competing Interests Statement.  Are there any patents, products in development or marketed products associated with this research to declare? If not, we would like to propose the following Competing Interests Statement to be published alongside your paper:   "CS is the recipient of the Sydney Partnership for Health, Education, Research and Enterprise (SPHERE) Palliative Care Clinical Academic Group Seed Grants. RC receives consulting fees or payments from Cymra Life Sciences Limited, Tilray Australia and New Zealand, AstraZeneca Pty Limited, A. Menarini Australia Pty Ltd. RC also receives payments for expert testimony from Office of the Director of Public Prosecutions NSW. AB assists in running a tertiary referral ketamine service at Black Dog Institute, running the Spravato (intranasal esketamine) early access program, and acting as a site principal investigator on a related quality of life study. CL participated in Janssen Advisory Board, acted as an unpaid consultant for Douglass Pharmaceuticals, and received support from Royal Australian and New Zealand College of Psychiatrists (RANZCP) for presenting at RANZCP Congress. DC is a paid consultant and advisory board member for Helsinn Pharmaceuticals, a paid consultant for Mayne Pharma International Pty Ltd, a paid subcontractor for Nous Group Pty Ltd, a paid board member for Icare Dust Diseases Board, and unpaid consultants for Chris O’Brien Lifehouse and Illawarra Health and Medical Research Institute (IHMRI). DC also receives payment from Mayne Pharma International Pty Ltd for intellectual property. Other authors declare no conflict of interest. This does not alter our adherence to PLOS ONE policies on sharing data and materials. There are no patents, products in development or marketed products associated with this research to declare.”  Please let us know if the above statement is accurate. | There are no patents, products in development or marketed products associated with this research to declare. The suggested wordings has been added in the track-changed version of the manuscript v1.9. |
|  |  |  |

Once again, thank you for the opportunity to address these issues.

Yours sincerely

Wei Lee

(On behalf of the authors)
